# Effects of 12 weeks of neuromuscular electrical stimulation of the quadriceps muscles on the function and physio-biochemical traits in functionally fit female nursing-home residents aged 75 + years: a pilot study

**DOI:** 10.1007/s00421-023-05321-1

**Published:** 2023-09-26

**Authors:** Ryszard Zarzeczny, Agnieszka Nawrat-Szołtysik, Anna Polak

**Affiliations:** 1 Institute of Health Sciences, Collegium Medicum, Jan Kochanowski University, 5 Żeromskiego Str., 25-369 Kielce, Poland; 2 Chair of Physiotherapy Basics, Jerzy Kukuczka Academy of Physical Education in Katowice, 72A Mikołowska Str., 40-065 Katowice, Poland

**Keywords:** Neuromuscular electrical stimulation, Quadriceps muscles strength, Physio-biochemical traits, Functional capability, Aged women

## Abstract

**Purpose:**

Muscular changes induced by neuromuscular electrical stimulation (NMES) are well recognized, but knowledge of how NMES influences the physio-biochemical traits of the oldest old is still limited. This study investigated the effect of NMES applied for 12 weeks to the quadriceps muscles of female nursing-home residents aged 75 + on their functional capability and inflammatory, bone metabolism, and cardiovascular traits.

**Methods:**

Nineteen women regularly taking part in two body conditioning sessions per week were randomized into an electrical stimulation group (ES; *n* = 10; 30 min sessions, 3 times per week) or a control group (CON; *n* = 9). At baseline and study week 12, all women performed the 30 s chair stand test (30sCST), the 6-minute walk test (6MWT), and the instrumented timed up and go test (iTUG). Resting heart rates, blood pressure, and the blood concentrations of inflammatory and bone metabolism markers were also measured twice.

**Results:**

NMES increased the strength of participants’ quadriceps muscles and their performance on the 30sCST and 6MWT while lowering resting arterial blood pressure and inflammatory marker levels; osteoclast activity showed a tendency to decrease. Changes in the iTUG results were not observed. A multiple regression analysis found that the results of functional tests in the ES group were best correlated with pulse pressure (the 30sCST and iTUG tests) and diastolic blood pressure (the 6MWT test).

**Conclusion:**

Twelve weeks of NMES treatment improved participants’ functional capacity and inflammatory, bone metabolism, and cardiovascular traits. The ES group participants’ performance on functional tests was best predicted by hemodynamic parameters.

## Introduction

Human populations are aging all over the world. The World Health Organization’s report “Decade of Healthy Ageing: Baseline Report” (WHO 2020a) estimates that the number of people aged 60 years and older will increase between 2019 and 2030 from 1 billion to 1.4 billion, and their percentage of the global population will reach 34%. The number of 80 years old and older people is increasing particularly fast, from 54 million in 1990 to 143 million in 2019. These demographic changes will soon bring on more socio-economic challenges related to the deteriorating health of aging populations**.**

Aging affects the functioning of most organs in the human body and impairs the efficiency of homeostatic mechanisms (Clegg et al. 2013). These changes are usually attributed in the literature to low-grade chronic inflammation (Marzetti et al. 2014; Arai et al. 2015; Chung et al. 2019), because it causes cumulative damage to tissues that deteriorates their mechanical properties and function (Lopez-Otin et al. 2013). Research has found close relationships between the serum level of inflammatory cytokines and seniors’ increased risk of cardiovascular diseases (Arnold et al. 2021), osteoporosis (Mundy 2007), sarcopenia (Dalle et al. 2017), physical disability (Penninx et al. 2004), and death (Harris et al. 1999).

According to available evidence, regular physical exercise can considerably mitigate the adverse effects of age-related physiological changes (see Garatachea et al. 2015). Conventional physical training is reported to be able to considerably reduce the blood concentration of inflammatory biomarkers (Woods et al. 2012; Timon et al. 2021) and arterial blood pressure (Sardeli et al. 2020) in older adults, as well as improving their lipid profiles (Sardeli et al. 2021) and bone metabolism (Pinheiro et al. 2020). However, many older adults fail to engage in the level of exercise recommended by WHO (2020b), partly because physical activity tends to decrease with age (Milanović et al. 2013) and partly due to poor health, fear of falling, or lower self-esteem (Baert et al. 2011). In such cases, neuromuscular electrical stimulation (NMES) seems a useful option, all the more so because research shows that this exercise technology is time-saving, joint-friendly, well-tolerated, and fun for older people (Kemmler et al. 2017).

NMES causes muscles to contract by activating muscle tissue and/or nervous cells (Lake 1992). The literature describes two main types of NMES: whole-body electromyostimulation (WB-EMS) and local NMES (Álvarez-Barrio et al. 2023). WB-EMS is delivered using electrodes attached to the opposite sides of the body and stimulates the agonist and antagonist muscles at the same time (Filipovic et al. 2012). The treated muscle area can be as large as 2800 cm^2^ (Kemmler and von Stengel 2017). Local NMES is used to stimulate a specific body site. A study in which athletes were allocated to receive WB-EMS and local NMES, respectively, for a period of 10 days to 14 weeks did not show gains in strength, muscle contraction velocity, or muscle power to be significantly different between the groups (Filipovic et al. 2012). Both WB-EMS and local NMES are well tolerated even by untrained people, but WB-EMS is considered a risk factor for exertional rhabdomyolysis (Stöllberger and Finsterer 2019), especially when misapplied (Kemmler et al. 2016).

As aging-related strength loss affects lower limbs more than upper body parts (Lynch et al. 1999), they should be a preferential target for rehabilitation in older adults, with NMES appearing to be particularly suitable for them. Research has shown that regular NMES sessions can increase the strength of the quadriceps muscles in the elderly (see Paillard 2018), delay muscle atrophy (Benavent-Caballer et al. 2014), stimulate anabolic processes while inhibiting catabolic processes in the muscles (Barberi et al. 2015), reduce the expression of muscle-specific atrophy-related ubiquitin ligase genes by increasing the expression of IGF-1 and relevant biomarkers of activated satellite cells and myoblasts, and to promote the remodeling of muscle fibers (Kern et al. 2014). NMES has also been found to improve performance on functional tests such as the 6-minute walk test (6MWT), timed up and go test (TUG), gait speed over the distance of 4 m, and standing balance and chair rising test (Caulfield et al. 2013; Cvecka et al. 2015; Zampieri et al. 2015; Kern et al. 2014). It is also reported to decrease the risk of aged subjects falling and enhances their quality of life (Langeard et al. 2017).

Studies investigating the effect of NMES on the strength of the lower limb muscles in elderly people are much greater in number compared with works analyzing the influence of NMES on the physiological and biochemical factors involved in age-related health problems, including chronic low-grade inflammation, osteoporosis, and cardiovascular changes (Paillard 2018). Conventional physical training has been found to reduce the level of pro-inflammatory cytokines in older people (Woods et al. 2012; Timon et al. 2021), but the ability of regular muscle contractions induced by NMES (without involving the nervous system) to produce the same effect is yet to be established.

With regard to bone metabolism, a 6-week training combining stairs climbing and quadriceps NMES has not significantly affected bone mineral density, but its increase was always located at the sites being submitted to strong mechanical constraints (e.g., femoral trochanter) (Paillard et al. 2003). In Arija-Blázquez et al. (2013), a bout of quadriceps NMES significantly lowered the levels of C-terminal crosslinking telopeptides of type I collagen (CTX-I) in patients with thoracic spinal cord injuries. Therefore, knowing that there are biochemical cross-talks between muscle and bone (Karsenty and Olson 2016) and assuming that NMES is capable of reducing the inflammatory level, the question arises whether regular NMES sessions can significantly influence the blood concentrations of the bone metabolism markers.

Finally, although NMES is known to preferentially target type II muscle fibers (Paillard et al. 2005), thus increasing the intramuscular concentration of anaerobic metabolites and significantly improving blood flow in the stimulated muscles in young people (Cramp et al. 2000), which is accompanied by repeatable shear stress (Davies et al. 2005), the cardiovascular response to NMES in older people who usually show blood vessel stiffness has not yet been described (Lan et al. 2023).

Considering the above findings, literature gaps, and the variety of age groups participating in NMES studies (the youngest participants were 60 years old), this study sets out to analyze the effect of 12 weeks of quadriceps NMES on the functional capability and physio-biochemical traits of female nursing-home residents aged 75 + . The following hypotheses were tested: (1) NMES increases the strength of the quadriceps muscles; (2) NMES improves performance on functional tests; (3) NMES reduces the blood concentration of inflammatory biomarkers; (4) NMES influences bone metabolism by increasing the activity of osteoblasts and diminishing the activity of osteoclasts; 5) NMES reduces blood pressure.

## Methods

### Participants

Nineteen women aged 75 and older were recruited from the residents of nursing homes in the Polish region of Upper Silesia. They were selected based on their medical histories, interviews, and physical examinations. The inclusion criteria required that they were aged 75 years or older, independent in performing basic activities of daily living (a score of 6 on the Katz Index of Independence in Activities of Daily Living (ADL) (Wallace and Shelkey 2008)), capable of logical verbal communication, and free of medical contraindications to exercise. The women’s ability to perform basic ADLs was assessed by the nursing-home nurses. Their physical fitness was evaluated by geriatricians based on medical histories, physical examinations, and the ability to walk 20 m without assistance or rest (Donat Tuna et al. 2009). Women were considered ineligible for the study if they had cancer, uncontrolled high blood pressure, atrial fibrillation, a cardiac pacemaker, amputations, epilepsy, neurodegenerative disorders, hyperthyroidism, hyperparathyroidism or malabsorption, used diuretics or walking aids, had a history of bone and mineral metabolism disorders (kidney, liver, thyroid and parathyroid glands diseases, malabsorption, and fractures suffered in the past 2 years), used medications influencing bone metabolism (e.g., glucocorticoids and anticonvulsants), were on bisphosphonate therapy for osteoporosis, hormone replacement therapy or calcitonin therapy within 1 year of screening, reported feeling joint pain at enrollment or in at least 1 month in the year preceding the study.

Prior to the study, the participants performed activities of daily living or were physically active for other reasons for less than 60 min per week (< 3 MET); additionally, they participated in conditioning training programs (CTP) provided by their homes’ staff.

Following enrollment, women were randomized into a control group (CON; *n* = 9) and an electrical stimulation group (ES; *n* = 10). Both groups participated in a 12-week CTP, which, in the ES group, was supplemented with 12 weeks of NMES.

Before they were recruited, all women signed informed consent forms and were advised on the study’s purpose and risks. The study design conformed to the internationally accepted policies governing the use of human subjects in research and was approved by the Bioethics Committee at the Jan Długosz University in Częstochowa, Poland (KB-2/2015; 7 May 2015). The authors did not have competing interests that might in any way affect the content of this article.

### Study design

The following procedures were carried out at baseline and repeated after 12 weeks. Blood samples were collected from fasted participants from 7.00 a.m. to 9.00 a.m., after which anthropometric, body composition, resting heart rate (HR), and arterial blood pressure (BP) measurements were performed. The measurements of isometric knee extension peak torque (IKEPT) and standard functional tests [the instrumented Timed Up and Go Test (iTUG), the 30 s Chair Stand Test (30sCST), and the 6-Minute Walk Test (6MWT)] were performed between 10.00 a.m. and 12.00 a.m. Participants were familiarized with IKEPT measurements and the tests 2 or 3 days before they took place. In preparation for IKEPT measurements and the 30sCST and iTUG tests, they did a warm-up consisting of a long-distance corridor walk and stretching exercises. Functional tests were conducted once daily in a random order over three consecutive days in similar ambient conditions (20–22 °C at 40–50% humidity), with strong verbal encouragement from the investigators. The tests were supervised by experienced physical therapists and the measurement equipment was operated by a qualified engineer.

Participants were instructed to avoid strenuous physical activity 1 day before the functional tests and to consume all drinks and liquid meals (containing an equivalent of ca 1500 ml of water; the daily energy intake was 1900–2000 kcal, with carbohydrates, fats, and proteins accounting for 63%, 24%, and 13%, respectively); they were served at the nursing home, starting 3 days before the study and continuing for 12 additional weeks.

### Procedures

Anthropometric measurements included body height (measured to the nearest 0.5 cm using a fixed stadiometer) and body mass (assessed to 0.1 kg using a body composition analyzer). The participants were lightly clothed and barefoot for both measurements.

Body composition assessments were conducted using the single-frequency (50 kHz, 90 µA) bioimpedance analyzer Tanita BC 420MA (Japan), which meets the requirements of the 93/42 EEC Directive for medical devices and whose usefulness has been confirmed by previous research (see Baran et al. 2022; Zarzeczny et al. 2018).

Resting heart rate (HR_rest_) and blood pressure (BP) were measured on sitting participants after allowing them 15 min of rest to avoid confounders such as previous exertion or anxiety with a fully automatic blood pressure monitor (Microlife BP A150, Microlife AG, Switzerland). Using the systolic (BP_syst_) and diastolic (BP_diast_) blood pressure values, pulse pressure (PP) (BP_syst_ – BP_diast_), and mean blood pressure (BP_mean_) (BP_diast_ + 1/3rd of the PP) were calculated.

IKEPT measurements were performed isometrically with a dynamometer (Jupiter, AC International East, Poland). Participants’ dominant legs were determined using the ball-kicking test (Springer et al. 2007). The women were asked to sit in the knee extension chair with straight back and the knee and hip joints at 90° of flexion, so that the lever arm of the extensor chair touched their ankle above the malleoli (Silanpӓӓ et al. 2014). On command “go”, they were to exert maximal isometric force against the lever arm. The torque was being displayed for them on the PC monitor to encourage them to push harder. Each participant performed three trials for each leg, with 90-s intervals between consecutive trials. The highest IKEPT achieved was included in analysis (Silanpӓӓ et al. 2014).

The iTUG test required the women to rise from a 46-cm high chair without armrests and walk as fast as they could to a line marked out on the floor 3 m away, turn around, walk back to the chair, and sit down again (Greene et al. 2014). Before the test was performed, they were allowed to walk through it once, so that they could familiarize themselves with it. When performing the test, the participants were wearing elastic belts with inertial sensing units (70 × 40 × 18 mm; 37 g; G-Sensor^®^, BTS Bioengineering S.p.A., Italy) at the level of lumbar segment L5. The units contained a tri-axial accelerometer and tri-axial gyroscope sensors (1000 Hz sample frequency) that communicated with the computer via Bluetooth. The test completion times were calculated by pre-installed software (BTS G-Walk^®^). Collado-Mateo et al. (2019) have confirmed the reliability of the iTUG, finding its intraclass correlation to be 0.903.

The 30sCST required the subjects to rise from a 46-cm high chair without armrests and sit back down as many times as they could within 30 s (Millor et al. 2013). According to Collado-Mateo et al. (2019), the test has intraclass correlation of 0.874, which confirms its reliability.

The 6MWT required the participants to walk a maximum distance during 6 min, slowing down or taking a rest when needed (Enright et al. 2003). The test was performed in line with the safety recommendations by the American Thoracic Society statement (American Thoracic Society Committee on Proficiency Standards for Clinical Pulmonary Function Laboratories 2002). Its reliability has been confirmed by the Rikli and Jones (1998), who estimated its intraclass correlation at 0.895.

### Conditioning training program (CTP)

Identically structured conditioning exercise sessions of 45 min were performed under the supervision of physical therapists on Tuesdays and Thursdays between 10.00 and 12.00 a.m., 2 h after a breakfast, in similar ambient conditions (20–22 °C at 40–50% humidity). The participants exercised while sitting on chairs or balance discs. A 5-min warm-up involving pedaling on rehabilitation rotors (ASEWUN DBT-X002, China) was followed by a 15-min bout of stretching, balance, and coordination exercises with balance discs and sticks. The next 20 min were allocated to 8–10 strength exercises engaging the major muscle groups (upper and lower extremity muscles, chest muscles, and back and abdominal muscles), which the participants performed using training weights, elastic bands (Theraband Latex Resistance Bands), and body resistance. The initial mass of the weights was 0.5 kg and was increased every 3 weeks by 0.5 kg. Each exercise was repeated 10–15 times. Repetitions lasted 3 s and were separated by rest intervals of 90 s. The sessions concluded with a 5-min warm-down consisting mostly of breathing exercises. The intensity of exercise was maintained at 60% HRR (heart rate reserve).

The exercises were intended to improve participants’ cardiorespiratory fitness and neuromuscular coordination, and prevent the development of contractures, degenerative joint disease, and muscle atrophy.

### Neuromuscular electrical stimulation (NMES) protocol

**Fig. 1:**
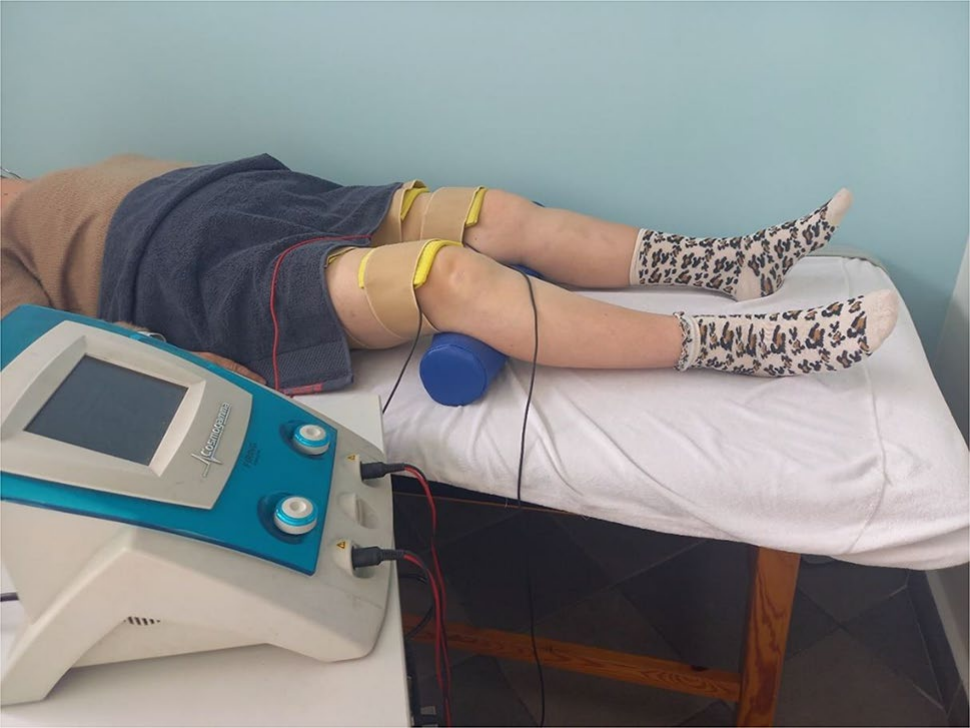
The setup of an NMES session

**Fig. 2:**
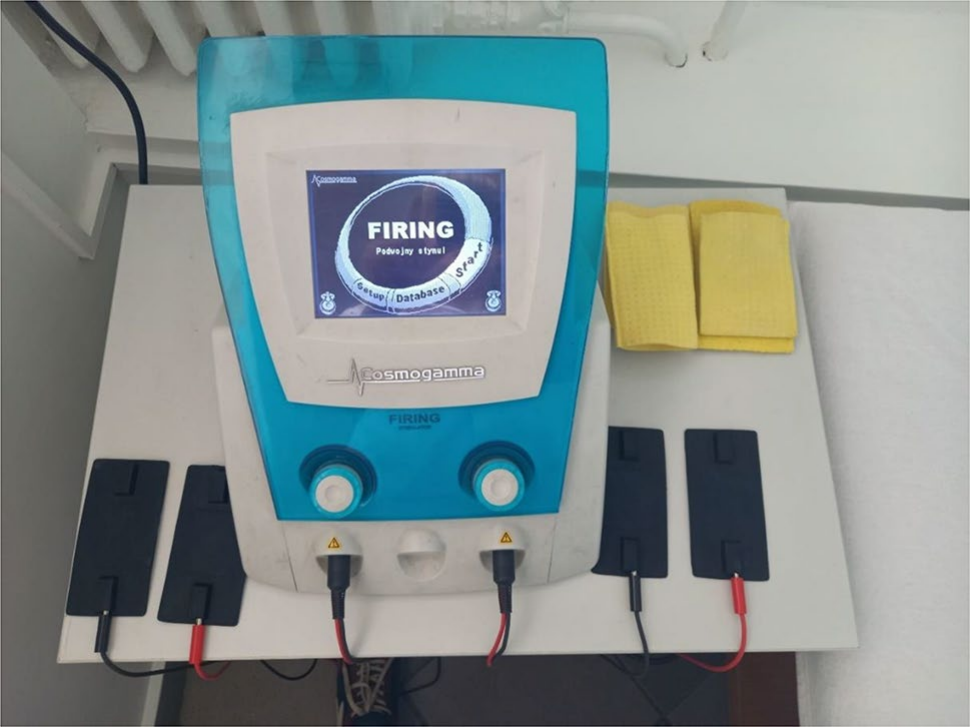
The NMES equipment used in the study

Thirty-minute NMES sessions supervised by physical therapists were performed between 10.00 and 12.00 a.m. (2 h after breakfast) once daily, three times per week (Mondays, Wednesdays, and Fridays) for 12 weeks, in similar ambient conditions (20–22 °C, 40–50% humidity). When receiving NMES, the participants were laying in the supine position, with the legs relaxed and slightly flexed at the hip, and the knee joints flexed at 20°–30° [the knees were supported for comfort by an appropriately high roll (Fig. 1)]. NMES was delivered using two pairs of 10 cm × 15 cm carbon rubber electrodes (150 cm^2^), one for each thigh, placed 4 cm below the anterior superior iliac spine and 4 cm above the patella and held in position by straps. Moist pads (12 cm × 17 cm; 204 cm^2^) separated them from cleansed skin. The pairs of electrodes were connected to a two-channel stimulator (Firing Evo, SNF 7457 Cosmogamma, Italy) and independently and simultaneously stimulated both quadriceps muscles (Fig. 2). Biphasic rectangular pulses (pulse-width of 0.3 ms, between-pulse interval of 33 ms; period 0.3 ms + 33.3 ms; duty cycle: 0.3 ms: 33.3 ms = 0.009; 0.9%) and a 30 Hz current frequency were used. ON and OFF times were 2 and 4 s (duty cycle = 2 s: 6 s = 0.33; 30%). NMES intensity was patient selected, so that it caused visible but well-tolerated painless contractions of the quadriceps muscles (current intensity was being increased until an upward jerk of the heel when it was reduced by 5 mA). Incomplete tetanic contractions of the quadriceps muscles were observed during treatment. This NMES protocol was designed based on Paillard’s study (2018) with older subjects.

### Blood sampling and biochemical assays

Venous blood samples (10 ml) were collected by a qualified nurse at baseline and at intervention week 12 between 7 and 9 a.m., after a 12-h fast. Samples were left to coagulate at room temperature, and then, they were centrifuged for 10 min at 1000×*g* to separate serum. One part of serum from each sample was assayed on the same day using a p-nitrophenyl phosphate photometric method (utilizing a fully automated system COBAS C 311 analyzer, Roche Diagnostics, Switzerland) to determine the activity of total alkaline phosphatase (TAP; the laboratory normal range of TAP is 44–147 U/l). The other part was stored at − 80 °C to measure the activity of bone-specific alkaline phosphatase (BAP) and the concentrations of C-terminal crosslinking telopeptides of type I collagen (CTX-I), interleukin-6 (IL-6), and tumor necrosis factor alpha (TNF-α). All assays were duplicated.

The absolute activity of BAP was determined using a heat-inactivation method (Kubo et al. 2012; the laboratory normal range of BAP is 14.2–54.8 U/l)*.* An intra-assay coefficient of variation (CV) of BAP was calculated to be 4.15%. The percentage of BAP activity (%BAP) was calculated in relation to TAP.

The serum level of CTX-I was measured with an enzyme-linked immunosorbent assay (ELISA) (Cat. No. AC-02F1, Immunodiagnostic Systems, UK; the laboratory normal range of CTX-I is 0.1–1.3 ng/ml). The intra-assay CV calculated for CTX-I was 3.29%. Serum high-sensitivity IL-6 and TNF-α were assayed using 96-well ELISA plates (Diaclone, France) as per the manufacturer’s instructions (Cat. No. 950.035.096 and 950.090.096, respectively). The laboratory normal ranges of IL-6 and TNF-α are 0.8–4.7 pg/ml and < 8 pg/ml, respectively. The intra-assay CVs for IL-6 and TNF-α were 5.73% and 7.88%. All biochemical analyses were performed by a highly qualified laboratory technician.

### Statistical analysis

The normality of data distribution was verified by the Shapiro–Wilk test. Data with non-normal distributions were transformed into logarithms for further analysis. The significance of differences between groups’ characteristics were determined by the Student t test for unpaired samples.

The statistical significance of between-group differences in the selected variables was determined pre- and post-intervention using a two-way, repeated-measures analysis of variance (ANOVA) with one factor (time). The post hoc analysis of ANOVA results was conducted using the Neumann–Keuls test. ANOVA was also used to calculate the partial effect sizes of particular traits. Correlations between the results of functional tests and participants’ physio-biochemical traits were presented as Pearson’s product–moment correlation coefficients. To prevent the occurrence of type-1 errors associated with multiple comparisons, the Benjamini–Hochberg procedure and a False Discovery Rate of 0.15 were used, as proposed by McDonald (McDonald 2014). Each variable’s contribution to the results of functional tests was estimated using a stepwise multiple regression analysis with backward elimination. All analyzed variables were significantly correlated with the dependent variable. The computations were performed in Statistica 12.0 (Statsoft, Poland).

The statistical analysis results were represented by arithmetic means and standard deviations (± SD). When their distributions were not normal, medians (*M*) and interquartile ranges (IQR) were used. Differences were considered significant at the level of *p* < 0.05 with a confidence level of 0.95, excluding multiple comparisons (the Benjamini–Hochberg procedure).

## Results

The study was conducted with 19 women [mean age 83.6 ± 5.5 years (75–93 years); mean body height 1.54 ± 0.07 m (1.41–1.65 m), mean body mass 63.2 ± 10.5 kg (44.5–88.6 kg), and mean body mass index (BMI)—26.56 ± 4.52 kg/m^2^ (18.52–35.11 kg/m^2^)]. Ten of them (52.6%) were treated for hypertension, 5 (26.3%) for diabetes, and 9 (47.4%) used antidepressants. The baseline comparability of the ES and CON groups (Table 1) was confirmed by ANOVA (Tables 2, 3, 4, 5).

**Table 1:** Baseline descriptive statistics of the study participants (ES—electrical stimulation group, *n* = 10; CON—control group, *n* = 9)

Variable	CON	ES	*p*
Age (years)	83.889 ± 4.400 Range: 75–91	83.400 ± 6.501 Range: 75–93	0.852 n.s.
Body height (m)	1.528 ± 0.068 Range: 1.42–1.62	1.569 ± 0.067 Range: 1.41–1.65	0.313 n.s.
Body mass (kg)	60.933 ± 7.309 Range: 50.1–69.6	65.210 ± 12.736 Range: 44.5–88.6	0.389 n.s.
BMI (kg/m^2^)	26.222 ± 3.676 Range: 21.1–32.5	26.904 ± 5.355 Range: 18.5–35.1	0.753 n.s.
*Use of drugs*			
Anti-hypertensive (n)	5 (55.6%)	5 (50.0%)	–
Anti-diabetic (n)	2 (22.2%)	3 (30.0%)	–
Anti-depressant (n)	5 (55.6%)	4 (40%)	–

The data represent means (± SD)

**Table 2:** Pre- and post-intervention values of the investigated somatic variables as generated by two-way ANOVA (ES—electrical stimulation group (*n* = 10); CON—control group (*n* = 9); *ɳ*^2^—partial effect size]

Variable		Pre-	Post-	Group effect	Time effect	Interaction effect
Body mass (kg)	CON	60.933 ± 7.309 Range: 50.1–69.6	61.222 ± 7.411 Range: 51.3–73.3	*F* = 0.716 n.s. *η*^2^ = 0.040	*F* = 0.056 n.s. *η*^2^ = 0.003	*F* = 0.043 n.s. *η*^2^ = 0.002
	ES	65.210 ± 12.736 Range: 44.5–88.6	65.230 ± 13.342 Range: 43.3–91.2			
BMI (kg/m^2^)	CON	26.222 ± 3.676 Range: 21.1–32.5	26.411 ± 4.086 Range: 22.2–33.2	*F* = 0.069 n.s. *η*^2^ = 0.004	*F* = 0.065 n.s. *η*^2^ = 0.004	*F* = 0.212 n.s. *η*^2^ = 0.012
	ES	26.904 ± 5.355 Range: 18.5–35.1	26.850 ± 5.238 Range: 18.0–33.5			
Fat (%)	CON	31.722 ± 6.691 Range: 18.3–40.8	32.289 ± 6.821 Range: 22.5–43.7	*F* = 0.373 n.s. *η*^2^ = 0.021	*F* = 1.752 n.s. *η*^2^ = 0.093	*F* = 0.598 n.s. *η*^2^ = 0.034
	ES	33.010 ± 8.886 Range: 16.0–46.3	35.170 ± 8.220 Range: 17.4–46.4			
FFM (kg)	CON	41.289 ± 4.126 Range: 35.4–49.6	41.200 (3.900) Range: 37.0–51.8	*F* = 0.136 n.s. *η*^2^ = 0.008	*F* = 1.269 n.s. *η*^2^ = 0.069	*F* = 0.846 n.s. *η*^2^ = 0.047
	ES	39.700 (4.450) Range: 37.4–55.9	39.950 (0.500) Range: 35.8–58.3			

The data represent means (± SD) and medians (IQR)

*BMI* body mass index, *FFM* fat-free mass

**Table 3:** Pre- and post-intervention results from two-way ANOVA for functional test performances [ES—electrical stimulation group (*n* = 10); CON—control group (*n* = 9); *η*^2^—partial effect size]

Variable		Pre-	Post-	Group effect	Time effect	Interaction effect
Dominant leg IKEPT (N m)	CON	25.561 ± 9.733 Range: 10.1–35.8	34.997^**^ ± 10.890 Range: 11.7–48.0	*F* = 10.544 *p* < 0.01 *η*^2^ = 0.383	*F* = 54.117 *p* < 0.001 *η*^2^ = 0.761	*F* = 4.812 *p* < 0.05 *η*^2^ = 0.221
	ES	46.286 ± 20.432 Range: 19.6–81.7	63.740^***a^ ± 22.157 Range: 33.7–100.8			
Non-dominant leg IKEPT (N m)	CON	27.570 (10.620) Range: 18.11–64.8	31.717 ± 7.276 Range: 16.46–41.0	*F* = 11.727 *p* < 0.01 *η*^2^ = 0.408	*F* = 17.446 *p* < 0.001 *η*^2^ = 0.506	*F* = 17.917 *p* < 0.001 *η*^2^ = 0.513
	ES	38.265 ± 9.923 Range: 17.0–56.0	68.654^***aa^ ± 23.546 Range: 33.0–113.7			
iTUG (s)	CON	25.800 ± 11.04 Range: 13.2–50.2	22.900 ± 10.887 Range: 10.9–46.7	*F* = 1.143 n.s. *η*^2^ = 0.063	*F* = 5.698 *p* < 0.05 *η*^2^ = 0.251	*F* = 0.031 n.s. *η*^2^ = 0.002
	ES	18.875 (7.400) Range: 13.2–40.7	18.470 ± 8.188 Range: 9.9–37.4			
30sCST (no. of repetitions)	CON	6.778 ± 2.017 Range: 4–11	8.667 ± 3.775 Range: 4–15	*F* = 0.808 n.s. *η*^2^ = 0.045	*F* = 12.919 *p* < 0.01 *η*^2^ = 0.432	*F* = 0.363 n.s. *η*^2^ = 0.021
	ES	7.500 ± 2.560 Range: 3–11	10.150^*^ ± 3.350 Range: 4–15			
6MWT (m)	CON	137.222 ± 70.275 Range: 51–275	118.222^*^ ± 51.603 Range: 35–210	*F* = 3.152 n.s. *η*^2^ = 0.156	*F* = 0.208 n.s. *η*^2^ = 0.012	*F* = 15.956 *p* < 0.001 *η*^2^ = 0.484
	ES	181.350 ± 98.228 Range: 70–385	205.250^**^ ± 92.339 Range: 90–400			

The data represent means (± SD) and medians (IQR)

*30sCST* 30-s chair stand test, *6MWT* 6-min walk test, *IKEPT* isometric knee extension peak torque, *iTUG* instrumented timed up and go test

*Significant pre-post difference within group (*p < 0.05; **p < 0.01; ***p < 0.001)

^a^Significant difference between the groups (CON-ES) (^a^*p* < 0.05; ^aa^*p* < 0.01; ^aaa^*p* < 0.001)

**Table 4:** Pre- and post-intervention results from two-way ANOVA for biochemical blood variables [ES—electrical stimulation group (*n* = 10); CON—control group (*n* = 9); *η*^2^—partial effect size]

Variable		Pre-	Post-	Group effect	Time effect	Interaction effect
IL-6 (pg/ml)	CON	6.323 ± 2.888 Range: 2.77–10.83	3.500 (1.930) Range: 2.16–10.82	*F* = 3.995 n.s. *η*^2^ = 0.190	*F* = 9.676 *p* < 0.01 *η*^2^ = 0.363	*F* = 0.248 n.s. *η*^2^ = 0.014
	ES	5.279 ± 3.252 Range: 2.18–11.33	2.703 ± 1.119 Range: 1.32–4.85			
TNF-α (pg/ml)	CON	13.970 ± 8.228 Range: 6.05–31.94	5.580^***^ (1.400) Range: 4.30–14.51	*F* = 1.329 n.s. *η*^2^ = 0.073	*F* = 33.722 *p* < 0.001 *η*^2^ = 0.665	*F* = 0.605 n.s. *η*^2^ = 0.034
	ES	10.157 ± 4.544 Range: 6.04–19.05	5.752^**^ ± 1.043 Range: 3.83–7.73			
CTX-I (ng/ml)	CON	0.378 ± 0.175 Range: 0.10–0.63	0.471 ± 0.296 Range: 0.10–0.96	*F* = 0.371 n.s. *η*^2^ = 0.021	*F* = 0.064 n.s. *η*^2^ = 0.004	*F* = 6.016 *p* < 0.05 *η*^2^ = 0.261
	ES	0.417 ± 0.313 Range: 0.10–1.02	0.302 ± 0.178 Range: 0.10–0.63			
TAP (U/l)	CON	96.189 ± 23.609 Range: 73.7–141.7	93.822 ± 20.184 Range: 69.2–133.9	*F* = 0.453 n.s. *η*^2^ = 0.026	*F* = 0.739 n.s. *η*^2^ = 0.042	*F* = 0.003 n.s. *η*^2^ = 0.000
	ES	90.790 ± 18.340 Range: 67.8–121.5	88.100 ± 13.473 Range: 66.5–107.0			
BAP (U/l)	CON	40.700 ± 11.771 Range: 23.7–64.2	44.278 ± 13.051 Range: 30.2–70.3	*F* = 0.009 n.s. *η*^2^ = 0.001	*F* = 0.790 n.s. *η*^2^ = 0.044	*F* = 0.445 n.s. *η*^2^ = 0.026
	ES	42.680 ± 12.420 Range: 27.1–70.8	43.190 ± 8.262 Range: 32.5–60.0			
BAP (%)	CON	42.710 (4.940) Range: 32.03–45.90	46.883 ± 6.722 Range: 36.43–56.54	*F* = 2.222 n.s. *η*^2^ = 0.116	*F* = 4.922 *p* < 0.05 *η*^2^ = 0.225	*F* = 0.364 n.s. *η*^2^ = 0.021
	ES	46.303 ± 6.127 Range: 37.80–58.27	49.166 ± 6.440 Range: 39.50–57.36			

The data represent means (± SD) and medians (IQR)

*BAP* bone-specific alkaline phosphatase, *CTX-I* C-terminal crosslinking telopeptides of type I collagen, *IL-6* interleukin-6, *TAP* total alkaline phosphatase, *TNF-α* tumor necrosis factor alpha

*Significant pre-post difference within group (*p < 0.05; **p < 0.01; ***p < 0.001)

^a^Significant difference between the groups (CON-ES) (^a^*p* < 0.05; ^aa^*p* < 0.01; ^aaa^*p* < 0.001)

**Table 5:** Pre- and post-intervention results from two-way ANOVA for cardiovascular variables [ES—electrical stimulation group (*n* = 10); CON—control group (*n* = 9); *η*^2^—partial effect size]

Variable		Pre-	Post-	Group effect	Time effect	Interaction effect
HR_rest_ (beats/min)	CON	70.111 ± 10.541 Range: 54–84	68.333 ± 12.874 Range: 44–87	*F* = 3.126 n.s. *η*^2^ = 0.155	*F* = 1.747 n.s. *η*^2^ = 0.093	*F* = 0.019 n.s. *η*^2^ = 0.001
	ES	79.000 (2.750) Range: 59–94	75.300 ± 9.129 Range: 60–93			
BP_syst_ (mm Hg)	CON	128.00 ± 14.603 Range: 100–154	126.444 ± 13.116 Range: 110–155	*F* = 0.684 n.s. *η*^2^ = 0.039	*F* = 5.342 *p* < 0.05 *η*^2^ = 0.239	*F* = 3.408 n.s. *η*^2^ = 0.167
	ES	138.800 ± 14.891 Range: 115–157	124.900^*^ ± 14.019 Range: 100–153			
BP_diast_ (mm Hg)	CON	69.444 ± 7.038 Range: 56–80	72.111 ± 8.085 Range: 60–80	*F* = 1.356 n.s. *η*^2^ = 0.074	*F* = 1.480 n.s. *η*^2^ = 0.080	*F* = 6.280 *p* < 0.05 *η*^2^ = 0.270
	ES	79.100 ± 12.297 Range: 60–95	71.400^*^ ± 9.336 Range: 60–85			
BP_mean_ (mm Hg)	CON	88.963 ± 7.978 Range: 76.7–102.0	90.200 ± 7.837 Range: 80.0–105.0	*F* = 1.578 n.s. *η*^2^ = 0.085	*F* = 3.056 n.s. *η*^2^ = 0.152	*F* = 5.133 *p* < 0.05 *η*^2^ = 0.232
	ES	99.000 ± 11.953 Range: 80.0–114.3	89.233^*^ ± 9.165 Range: 73.3–100.0			
PP (mm Hg)	CON	58.556 ± 13.492 Range: 35–78	54.333 ± 13.332 Range: 35–75	*F* = 0.001 n.s. *η*^2^ = 0.000	*F* = 9.177 *p* < 0.01 *η*^2^ = 0.351	*F* = 0.330 n.s. *η*^2^ = 0.019
	ES	59.700 ± 11.973 Range: 43–84	53.500 ± 13.352 Range: 40–84			

The data represent means (± SD) and medians (IQR)

*BP* blood pressure, *HRrest* resting heart rate, *PP* pulse pressure

*Significant pre-post difference within group (*p < 0.05; **p < 0.01; ***p < 0.001)

^a^Significant difference between the groups (CON-ES) (^a^*p* < 0.05; ^aa^*p* < 0.01; ^aaa^*p* < 0.001)

Neither during CTP sessions nor NMES treatments were incidents of medical significance recorded.

According to ANOVA, the between-group effect was significant only for the dominant and non-dominant legs’ IKEPT (*p* < 0.01 in both cases; Table 3). The dominant leg’s IKEPT increased between baseline and week 12 in both groups (CON *p* < 0.01 and ES *p* < 0.001), but post-intervention, its value was significantly greater in the ES group than in the CON group (*p* < 0.05). The baseline IKEPTs of the dominant and non-dominant legs were not significantly different between the groups (*p* > 0.05), but in the ES group, the non-dominant leg’s IKEPT increased significantly over the study (*p* < 0.001) and was significantly greater at week 12 compared with the CON group (*p* < 0.01) (Table 3). In the CON group, the baseline and week-12 IKEPTs of the non-dominant leg were not significantly different, but the dominant leg’s IKEPT changed significantly. Significant changes were also observed in the ES group in the results of the 30sCST (*p* < 0.05), the 6MWT (*p* < 0.01) (Table 3), the TNF-α level (*p* < 0.01) (Table 4), systolic BP (*p* < 0.05), diastolic BP (*p* < 0.05), and mean BP (*p* < 0.05) (Table 5); in the CON group, the results of the 6MWT (*p* < 0.05) (Table 3) and the blood concentrations of TNF-α changed significantly (*p* < 0.001) (Table 4).

The effect of time was significant for several variables. Significant increases were recorded for the dominant and non-dominant legs’ IKEPT (*p* < 0.001 in both cases), the 30sCST results (*p* < 0.01) (Table 3), and %BAP (*p* < 0.05) (Table 4), and significant decreases for the iTUG results (*p* < 0.05) (Table 3), IL-6 (*p* < 0.01), TNF-α (*p* < 0.001) (Table 4), systolic BP (*p* < 0.05), and PP (*p* < 0.01) (Table 5). In the ES group, the concentration of IL-6 showed a marked tendency to decline (*p* = 0.051), but it was insufficient for a statistically significant change to take place.

**Fig. 3:**
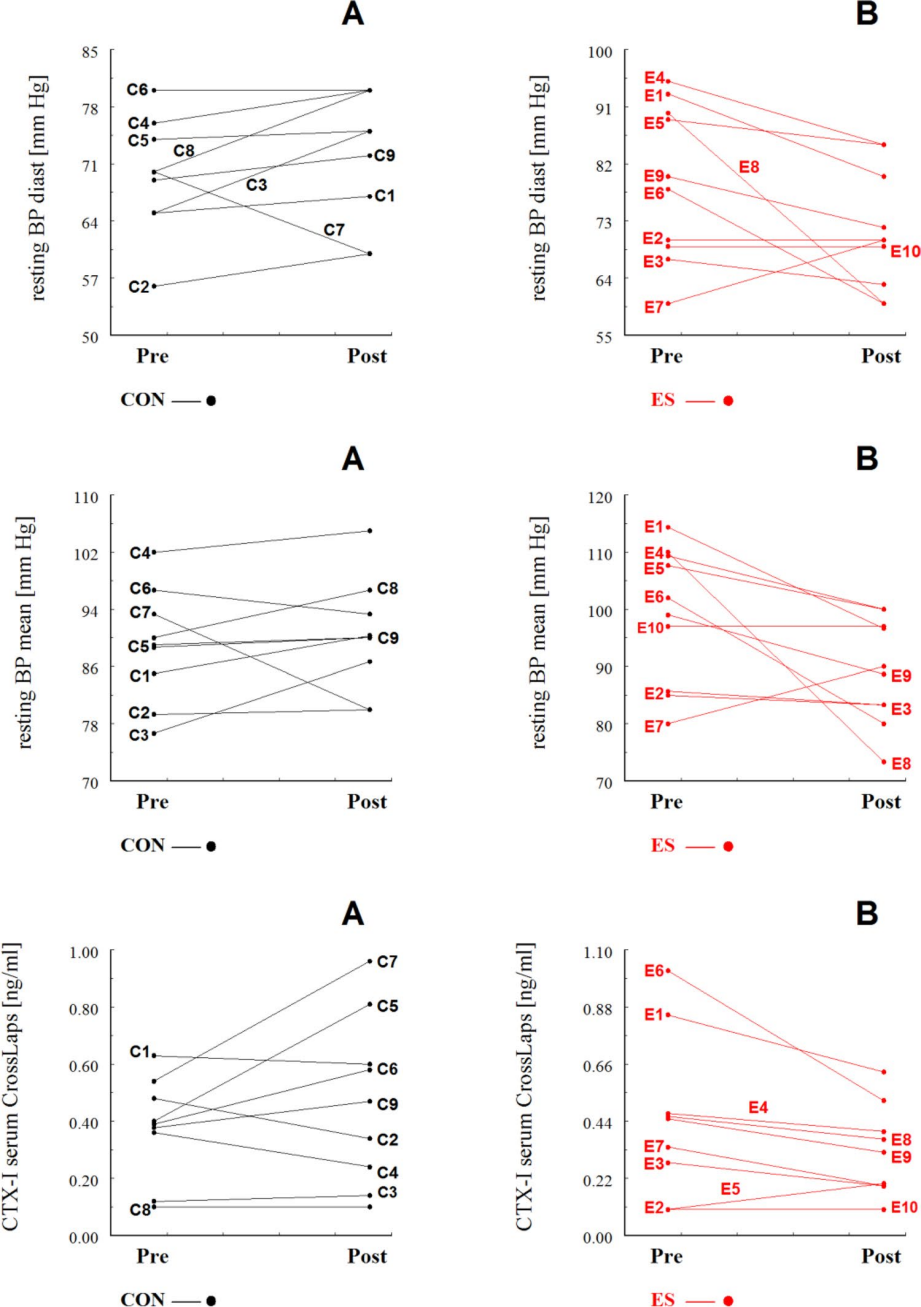
Individual values of resting diastolic blood pressure (BPdiast), resting mean blood pressure (BPmean) and -terminal crosslinking telopeptides of type I collagen (CTX) for control (**A**) and ES group (**B**). “C” and “E” represent a given subject in the control and experimental groups, respectively

**Fig. 4:**
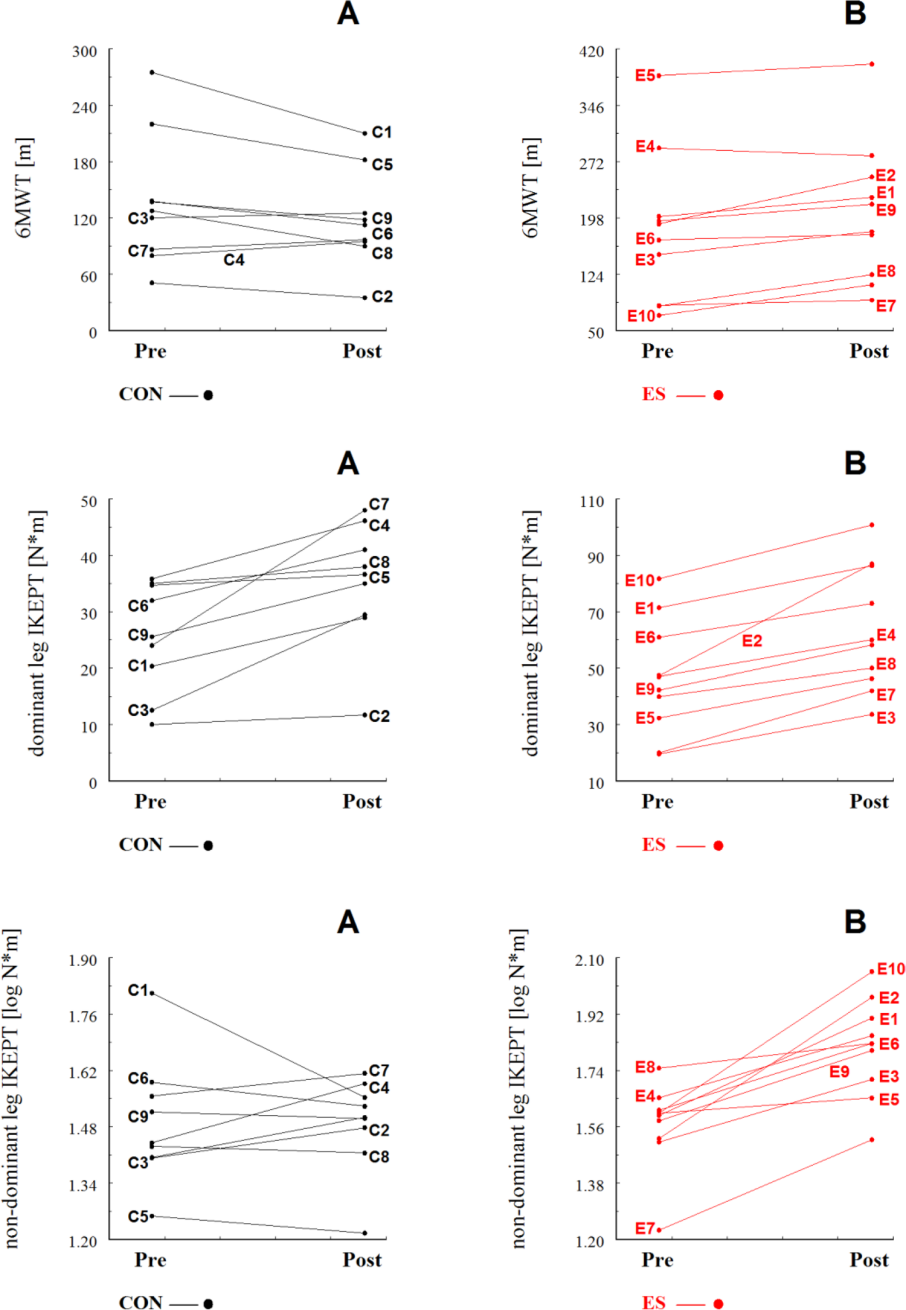
Individual values of the 6-min walk test (6MWT); dominant leg isometric knee extension peak torque (IKEPT) and non-dominant leg isometric knee extension peak torque (IKEPT) for control (**A**) and ES group (**B**). “C” and “E” represent a given subject in the control and experimental groups, respectively

The interaction effect was significant for six variables: the IKEPTs of the dominant leg (*p* < 0.05) and the non-dominant leg (*p* < 0.001), the results of the 6MWT (*p* < 0.001), CTX-I (*p* < 0.05), diastolic BP (*p* < 0.05), and mean BP (*p* < 0.05). Interactions for these six variables and individual participants in the ES and CON groups are shown in Figs. 3 and 4.

The Pearson product–moment correlation analysis showed that the results of functional tests were statistically significant and interrelated (Table 6). Although the IKEPTs of the dominant and non-dominant legs were significantly correlated with each other (*p* < 0.001), both were significantly correlated only with the results of the 6MWT (*p* < 0.05 in both cases); the dominant leg’s IKEPT was additionally correlated with the results of the 30sCST (*p* < 0.01). Neither IKEPT was related to the results of the iTUG (Table 6).

**Table 6:** The correlations between the results of functional tests and IKEPT values—all participants (*n* = 38)

Variable	Dominant leg IKEPT (N m)	Non-dominant leg IKEPT (N m)	iTUG (s)	30sCST (no. of repetitions)	6MWT (m)
Dominant leg IKEPT (N m)	X	0.612 *p* < 0.001	− 0.315 n.s.	0.430 *p* < 0.01	0.393 *p* < 0.05
Non-dominant leg IKEPT (N m)	0.612 *p* < 0.001	X	− 0.298 n.s.	0.303 n.s.	0.341 *p* < 0.05
iTUG (s)	− 0.315 n.s.	− 0.298 n.s.	X	− 0.693 *p* < 0.001	− 0.797 *p* < 0.001
30sCST (no. of repetitions)	0.430 *p* < 0.01	0.303 n.s.	− 0.693 *p* < 0.001	X	0.633 *p* < 0.001
6MWT (s)	0.393 *p* < 0.05	0.341 *p* < 0.05	− 0.797 *p* < 0.001	0.633 *p* < 0.001	X

*30sCST* 30-s chair stand test, *6MWT* 6-min walk test, *IKEPT* isometric knee extension peak torque, *iTUG* instrumented timed up and go test

Table 7 shows Pearson’s correlation coefficients between the physio-biochemical traits and the results of functional tests, adjusted for multiple comparisons by the Benjamini–Hochberg procedure, calculated for the ES group. The results of particular tests correlate with different numbers of physio-biochemical traits (iTUG and 6MWT: 4; 30sCST: 8), with only pulse pressure and BMI being significantly associated with the results of all three tests.

**Table 7:** Pearson’s correlation coefficients between the results of functional tests and physio-biochemical variables adjusted for a false discovery rate of 0.15—the ES group (*n* = 20)

variable	iTUG		Variable	30sCST		Variable	6MWT		Benjamini–Hochberg critical value
	*r*	*p*		*r*	*p*		*r*	*p*	
PP (mm Hg)	0.635	0.003 Significant	IL-6 (pg/ml)	− 0.651	0.002 Significant	BMI (kg/m^2^)	− 0.570	0.009 Significant	0.01
BMI (kg/m^2^)	0.578	0.008 Significant	BMI (kg/m^2^)	− 0.643	0.002 Significant	BP_diast_ (mm Hg)	0.545	0.013 Significant	0.02
FFM (kg)	0.498	0.026 Significant	PP (mm Hg)	− 0.565	0.009 Significant	PP (mm Hg)	− 0.536	0.015 Significant	0.03
BP_diast_ (mm Hg)	− 0.463	0.040 Significant	FFM (kg)	− 0.528	0.017 Significant	IL-6 (pg/ml)	− 0.531	0.016 Significant	0.04
Body mass (kg)	0.346	0.135 n.s.	BAP (U/l)	0.518	0.019 Significant	FFM (kg)	− 0.430	0.059 n.s.	0.05
IL-6 (pg/ml)	0.339	0.144 n.s.	Body mass (kg)	− 0.512	0.021 Significant	Body mass (kg)	− 0.395	0.085 n.s.	0.06
TNF-α (pg/ml)	0.231	0.326 n.s.	BAP (%)	0.464	0.039 Significant	BP_mean_ (mm Hg)	0.338	0.145 n.s.	0.07
HR_rest_ (beats/min)	− 0.226	0.338 n.s.	TNF-α (ph/ml)	− 0.425	0.062 Significant	BAP (%)	0.296	0.205 n.s.	0.08
BP_mean_ (mm Hg)	− 0.221	0.348 n.s.	TAP (U/l)	0.347	0.134 n.s.	HR_rest_ (beats/min)	0.262	0.264 n.s.	0.09
BP_syst_ (mm Hg)	0.180	0.447 n.s.	BP_syst_ (mm Hg)	− 0.295	0.207 n.s.	TNF-α (pg/ml)	− 0.232	0.325 n.s.	0.10
BAP (U/l)	− 0.176	0.459 n.s.	Fat (%)	− 0.283	0.227 n.s.	BAP (U/l)	0.166	0.486 n.s.	0.11
BAP (%)	− 0.170	0.474 n.s.	CTX-I (ng/ml)	0.279	0.234 n.s.	Fat (%)	− 0.135	0.570 n.s.	0.12
TAP (U/l)	− 0.125	0.601 n.s.	BP_diast_ (mm Hg)	0.224	0.342 n.s.	CTX-I (ng/ml)	− 0.065	0.786 n.s.	0.13
Fat (%)	0.047	0.844 n.s.	BP_mean_ (mm Hg)	0.013	0.958 n.s.	BP_syst_ (mm Hg)	− 0.042	0.861 n.s.	0.14
CTX-I (ng/ml)	− 0.015	0.949 n.s.	HR_rest_ (beats/min)	− 0.001	0.996 n.s.	TAP (U/l)	0.033	0.889 n.s.	0.15

*30sCST* 30-s chair stand test, *6MWT* 6-min walk test, *BAP* bone-specific alkaline phosphatase, *BMI* body mass index, *BP* blood pressure, *CTX-I* C-terminal crosslinking telopeptides of type I collagen, *FFM* fat-free mass, *HRrest* resting heart rate, *IL-6* interleukin-6, *iTUG* instrumented timed up and go test, *PP* pulse pressure, *TAP* total alkaline phosphatase, *TNF-α* tumor necrosis factor alpha

Considering that several correlations between the physio-biochemical traits and the ES group’s results on functional tests were significant, a stepwise multiple regression analysis was deployed to find out which trait was the best predictor of performance on each test. The results of the iTUG and 30sCST proved to be the most dependent on PP, which explained 40.3% and 31.9% of their variability, respectively. Diastolic BP was best correlated with the results of the 6MWT, explaining 29.6% of its variability (Table 8).

**Table 8:** The results of a stepwise multiple regression analysis with backward elimination for the results of functional tests as the dependent variables

Dependent variable	*r*^2^	SEE	Independent variable	*ß* ± SE of *ß*	*B* ± SE of *B*	*p*
iTUG (s)	0.403 *p* < 0.01	± 0.138	Intercept	–	0.778 ± 0.144	< 0.001
			PP	0.635 ± 0.182	0.009 ± 0.002	< 0.01
30sCST (no. of repetitions)	0.319 *p* < 0.01	± 2.717	Intercept	–	16.864 ± 2.834	< 0.001
			PP	− 0.565 ± 0.194	− 0.142 ± 0.049	< 0.01
6MWT (s)	0.296 *p* < 0.05	± 80.650	Intercept	–	− 145.005 ± 124.135	n.s.
			BP_diast_	0.544 ± 0.198	4.496 ± 1.632	< 0.05

*30sCST* 30-second chair stand test, *6MWT* 6-min walk test, *BP* blood pressure, *iTUG* instrumented timed up and go test, *PP* pulse pressure

## Discussion

The purpose of the study was to determine whether 12 weeks of quadriceps NMES would improve the functional capability of the oldest-old female nursing-home residents and influence the selected physio-biochemical traits.

In the treated participants (the ES group), significant improvement in the quadriceps strength, performance on the 30sCST and 6MWT tests, and lower blood pressure and TNF-α blood concentrations were observed post-intervention. Their CTX-I levels also improved more compared with controls. To our knowledge, this is the first study to investigate the effect of prolonged NMES on blood cytokine concentrations and hemodynamic parameters in elderly persons.

The current position statements and consensus guidelines for physical activity in older adults recommend a multi-modal exercise prescription encompassing aerobic, strengthening, balance, and flexibility training (Izquierdo et al. 2021), because single-modality training (e.g., aerobic training) does not improve strength or balance and may contribute to higher rates of falls and osteoporotic fractures in at-risk individuals (Sherrington et al. 2017*)*. The CPT in which all nursing-home residents participated was designed to include the above types of training. It was the same for the control group, which did not receive any other treatment, and for the experimental group, which also received NMES (creating an untreated group was not possible due to ethical reasons). The post-intervention analysis showed that in the control group, the CPT significantly reduced TNF-α levels, improved the isometric strength of the quadriceps muscles in participants’ dominant legs, and helped preserve the levels of bone metabolism biomarkers, cardiovascular traits, and performance on the 30sCST and iTUG (significant pre–post-differences were not observed). Regarding the controls’ performance on the 6MWT, it was considerably worse 12 weeks of conditioning training, and their cardiorespiratory fitness was lower than at baseline. The reason for the decline occurred is difficult to explain. The weekly frequency of NMES sessions in this study (twice weekly) was not significantly different from the frequency of conditioning training sessions in other studies with the oldest-old people (Capanema et al. 2022). However, the position statements and consensus guidelines for physical activity in older adults explain that multicomponent exercise training performed at least 2 days a week increases these adults’ functional ability most effectively when accompanied by aerobic activities of at least moderate intensity performed three or more times per week during sessions of 30 to 45 min in length (Izquierdo et al. 2021). It is likely that our participants’ worse performance on the 6MWT after 12 weeks was caused by a smaller volume of aerobic training and/or inter-individual heterogeneity in physiological responses and adaptions to exercise training in “responders”, “non-responders”, and “adverse responders” (Gronwald et al. 2020).

Our NMES protocol drew on the protocol recommended for older adults (Paillard 2018). Of special importance in designing NMES protocols is the current’s frequency (30 Hz in our study) due to its association with muscle fatigability (Jones et al. 1979)*.* For young people, a frequency of 50–100 Hz is usually recommended (Filipovic et al. 2011). There is evidence that stimulation at 80 Hz accelerates muscle fatigue, but a frequency of 20 Hz does not have an adverse effect on muscle strength (Narici et al. 1991). The results of the cited studies also suggest that low-frequency stimulation can influence muscular endurance (Paillard et al. 2005). Given that, and knowing that aging muscles become weaker, slower, and tetanize at lower fusion frequencies (Narici et al. 1991), we selected a frequency lying in the middle between a frequency increasing muscular strength and a frequency improving endurance.

The comprehensive review of studies by Langeard et al. (2017) showed that their authors frequently reported improvements in the strength of lower limbs following the application of NMES. In our study, too, the muscle strength of both lower limbs increased significantly in the ES group between baseline and week 12, supporting hypothesis 1. Attributing this change to the influence of NMES seems rational, even though the IKEPTs of controls’ dominant legs also increased significantly, probably due to their dominant legs working harder during the CTP than the non-dominant legs. The fact that FFM and the percentage of fat were not significantly different between the groups points out that the main cause of greater muscle strength of the lower limbs in the ES was neuromuscular adaptation enhancing the so-called “muscle quality” (Pinto et al. 2014).

Studies on the effect of NMES on older people have shown its ability to improve their performance on functional tests (Langeard et al. 2017). This finding is consistent with our results showing that the ES group performed better on the 30sCST and the 6MWT post-intervention. Nevertheless, hypothesis 2 is only partially confirmed, because the group’s performance on the iTUG did not change statistically significantly over the intervention period. In the study by Jang and Park (2021), the TUG performance of the group that only did strengthening exercises did not significantly improve after 4 weeks of intervention, but the group that additionally received NMES needed significantly less time to complete the test. These results are different from ours, probably because the NMES protocol and CTP program used by Jang and Park (2021) were different from those we used and because their intervention ended after 4 weeks. It is also noteworthy that Jang and Park (2021) stimulated thigh muscles (vastus medialis and vastus lateralis) and used handgrip strength as a measure of participants’ general muscle strength.

Our previous study (Zarzeczny et al. 2017) with the oldest-old sedentary female residents of nursing homes did not find significant correlations between their IKEPTs and performance on the iTUG, 30sCST, and 6MWT either. The ES group performed better on the 30sCST and 6MWT tests after 12 weeks, mainly because the strength of their quadriceps muscles treated with NMES increased; the confirmation of this is significant correlations between both tests’ results and participants’ IKEPTs. A lack of correlation between the strength of the quadriceps and iTUG completion times can be explained in terms of their greater dependence on muscle power than on muscle strength (Zarzeczny et al. 2017). Studies on community-dwelling older people (Bean et al. 2002) and the residents of a chronic care hospital (Bassey et al. 1992) have demonstrated that the iTUG requires lower limb muscle power rather than muscle strength.

In our study, the concentrations IL-6 and TNF-α decreased between baseline and week 12 in both groups (a significant time effect), but according to post hoc analysis, only the reduction in the level of TNF-α was statistically significant. This result is in agreement with hypothesis 3. However, it is difficult to credibly explain, especially since IL-6 can be synthesized and released to the circulation from working skeletal muscles (Fischer 2006), and its physiological role in maintaining inflammatory status depends on its concentration in blood. According to existing evidence, the normal concentration of IL-6 acts as anti-inflammatory mediator, but overproduction of IL-6 has a pro-inflammatory effect resulting in chronic inflammation (Gabay 2006). Truong et al. (2017) have demonstrated that a single 30-min NMES session can increase the concentration of IL-6 and decrease the concentration of TNF-α; however, according to the available literature, the effects of prolonged NMES intervention are yet not known (Paillard 2018; Sanchis-Gomar et al. 2019). Considering that NMES interventions evoke significant muscle contractions mimicking those induced by the conventional physical exercise, we can only presume that a significant time effect for IL-6 observed in this study was related to typical adaptation to physical training occurring in older individuals (see Fischer 2006).

The results of research on TNF-α are ambiguous. Studies with young, healthy subjects **(**Jahromi et al. 2014) have not found any evidence that physical training has a significant effect on the concentration of TNF-α, but studies involving older persons (Macêdo Santiago et al. 2018) and cardiac patients (Gielen et al. 2003) have demonstrated the ability of regular physical exercise to reduce the level of TNF-α. The results we obtained are similar to those concerning the elderly. The physiological mechanism behind them could be the negative relationship between the concentrations of IL-6 and TNF-α (Fischer 2006; Truong et al. 2017) and a putative anti-inflammatory role of IL-6 (Sanchis-Gomar et al. 2019), because the mean level of IL-6 at week 12 was below its upper normal value. However, because the pre–post-differences in the level of TNF-α occurred in both groups that did the same CTP, the changes brought about by NMES should be interpreted with caution.

Studies investigating the effect of NMES on bone markers are few. The only available data come from research with subjects with spinal cord injuries. In Arija-Blázquez et al. (2013), a bout of quadriceps NMES significantly reduced CTX-I levels. Their next study with the same NMES protocol applied for 14 weeks failed to find differences in the concentrations of CTX-I between the experimental group and the control group (Arija-Blázquez et al. 2014), but both of them were very small, consisting of only 5 and 3 subjects, respectively. In our study, too, the groups were not significantly different in the concentration of CTX-I, but the interaction effect between the groups’ CTX-I proved significant. This finding only partially supports hypothesis 4. The explanation of why the CTX-I levels did not change significantly in either group would instinctively be sought in the influence of TNF-α. According to research, TNFR-1, the key signaling receptor for TNF-α, activates the nuclear factor κB (NF-κB) (Jang et al. 2021) that inhibits osteoblast differentiation and/or increases the formation and survival of osteoclasts and osteoclast activity (Yin et al. 2019) through a complex signaling pathway (Jang et al. 2021). This physiological mechanism may have also occurred in our study, because significant decreases in TNF-α levels were observed in both groups. However, the interaction effect for CTX-I was significantly different between the groups, which can only be explained in terms of the influence of electric current and magnetic field. As electrical bone stimulation is known to promote osteogenesis in various experimental conditions (cell cultures, animals, and humans) (Cadossi et al. 2020; Tamaki et al. 2016), the significant interaction effect between the groups’ CTX-1 was presumably related to the greater expression of osteoprotegerin and a higher ratio between osteoprotegerin and the receptor activator of the nuclear factor κB ligand (RANKL) in the ES group (Qin et al. 2013).

During volitional muscle contractions, mechanical forces and increased concentrations of metabolic end-products stimulate type III and IV afferents in the muscles, significantly increasing the pressor response (Fisher and White 2004). DE Macedo et al. (2022) have observed that single session of leg NMES increases metaboreflex activation, and Santos et al. (2013) have recorded reduced vascular resistance and a limited increase in BP in subjects who received NMES before exercise. In our study, NMES significantly lowered BP in the ES group, thus confirming hypothesis 5. Even though BP measurements were taken at rest, the result can be attributed to reduced sensitivity of muscle nervous afferents as a result of systematically repeated muscle contractions. This is very likely given that NMES mainly affects muscle II fibers (Paillard et al. 2005) that are more dependent on anaerobic metabolism, leading to excess of anaerobic metabolism end-products (Sullivan et al. 1988).

The level of functional capability is primarily determined by the efficiency of the cardiovascular system (Forman et al. 2017). The moderate relationship between the strength of our participants’ quadriceps muscles and their performance on functional tests, probably related to the presence of controls, prompted us to examine correlations between the results of functional tests and physio-biochemical traits for the ES group alone. The analysis pointed out that participants’ performance on the tests was the most strongly associated with PP and BP_diast_. According to Franklin (2006), in young adults (< 50 years old), low BP_diast_ is indicative of increased stroke volume (SV), but in older adults (≥ 60 years old), it may be associated with ventricular-arterial stiffness. Franklin has observed (2006) that increasing PP and decreasing BP_diast_ can be surrogate measurements for increasing central elastic artery stiffness. Our study found, however, that although BP_diast_ was statistically significantly lower at week 12, PP did not change significantly over the intervention period. A significantly lower BP_mean_ (a surrogate measure of peripheral resistance) in the ES group at week 12 suggests that NMES may have reduced arterial stiffness, which is negatively associated with the cardiorespiratory fitness of older adults (Albin et al. 2020). Another plausible explanation is an increase in SV*.*

The study has several limitations. First, as all participants were elderly female residents of nursing homes, they may not be representative of their age group. Second, as a result of strict inclusion and exclusion criteria, all women in the study were relatively fit and healthy. Third, the results of the study should be interpreted taking account of the NMES protocol we used, because the physio-biochemical effects of NMES tend to vary depending on how it is applied. Finally, the small sample size requires caution in generalizing and interpreting our results.

## Conclusion

Our study has demonstrated that 12 weeks of quadriceps NMES can significantly lower the concentrations of inflammatory markers and resting arterial blood pressure in elderly female residents of nursing homes. It also showed a tendency to reduce osteoclast activity. With regard to participants’ functional capability, NMES significantly improved their performance on 30sCST and 6MWT, mainly by increasing the strength of their quadriceps muscles. Participants’ improved performance on all functional tests is best explained by changes in cardiovascular traits. Conditioning training did not prevent a decrease in the level of cardiorespiratory fitness in the control group. The results of the study may serve as a guidance for physiotherapists who seek the most effective ways to help older people stay healthy and independent.

## Data Availability

The datasets generated during and/or analyzed during the current study are available from the corresponding author on reasonable request.

## Abbreviations

6MWT: 6-Minute walk test
30sCST: 30S chair stand test
%BAP: Percentage of bone-specific alkaline phosphatase activity
ADL: Activities of daily living
ANOVA: Analysis of variance
BAP: Bone-specific alkaline phosphatase
BMI: Body mass index
BP: Blood pressure
BP_diast_: Diastolic blood pressure
BP_mean_: Mean blood pressure
BP_syst_: Systolic blood pressure
CON: Control group
CTP: Conditioning training program
CTX-I: C-terminal crosslinking telopeptides of type I collagen
CV: Coefficient of variation
ELISA: Enzyme-linked immunosorbent assay
ES: Electrical stimulation group
FFM: Fat-free mass
HR: Heart rate
HRR: Heart rate reserve
IKEPT: Isometric knee extension peak torque
IL-6: Interleukin-6
IQR: Interquartile range
iTUG: Instrumented timed up and go test
MET: Metabolic equivalent
NMES: Neuromuscular electrical stimulation
PP: Pulse pressure
SD: Standard deviation
TAP: Total alkaline phosphatase
TNF-α: Tumor necrosis factor alpha
WB-EMS: Whole-body electromyostimulation
WHO: World Health Organization
